# Long-Term Oncological Outcomes in Metastatic Prostate Cancer Patients Who Are Able to Maintain/Recover Ongoing Anticancer Therapy After SARS-CoV-2 Infection—Results of the MEET-URO 22 Study

**DOI:** 10.3390/cancers18020264

**Published:** 2026-01-15

**Authors:** Orazio Caffo, Umberto Basso, Antonello Veccia, Marco Maruzzo, Brigida Anna Maiorano, Consuelo Buttigliero, Claudia Mucciarini, Alessia Mennitto, Paola Ermacora, Mariella Sorarù, Maria Giuseppa Vitale, Cecilia Anesi, Dzenete Kadrija, Francesca Maines, Franco Morelli, Caterina Romeo, Davide Bimbatti, Isabella Saporita, Francesco Pierantoni

**Affiliations:** 1 Center for Medical Science, University of Trento, 38122 Trento, Italy; 2 Medical Oncology Department, Santa Chiara Hospital, APSS, 38122 Trento, Italydzenete.kadrija@apss.tn.it (D.K.);; 3 Oncology Unit 1, Istituto Oncologico Veneto, IOV IRCCS, 35128 Padova, Italy; 4 Oncology Unit 3, Istituto Oncologico Veneto, IOV IRCCS, 35128 Padova, Italy; 5 Oncology Unit, IRCCS Ospedale Casa Sollievo della Sofferenza, 71013 San Giovanni Rotondo, Italy; maiorano.brigida@hsr.it (B.A.M.); mfrancosilvia@libero.it (F.M.); 6 Department of Oncology, San Luigi Gonzaga Hospital, University of Turin, 10043 Orbassano, Italyisabella.saporita@unito.it (I.S.); 7 Oncology Unit, AUSL Modena, Ramazzini Hospital, 41012 Carpi, Italy; 8 Medical Oncology Department, “Maggiore della Carità” University Hospital, 28100 Novara, Italy; alessia.mennitto@maggioreosp.novara.it; 9 Oncology Department, S. Maria della Misericordia Hospital, 33100 Udine, Italy; 10 UOC Oncologia, AULSS 6 Euganea, General Hospital, 35012 Camposampiero, Italy; 11 Division of Oncology, Department of Oncology and Hematology, University of Modena, 41124 Modena, Italy

**Keywords:** metastatic castration-resistant prostate cancer, metastatic hormone-sensitive prostate cancer, prostate cancer, SARS-CoV-2 infection

## Abstract

**Background:** Although the relationship between androgen deprivation therapy (ADT) for prostate cancer (PC) and the biological mechanisms of severe acute respiratory syndrome coronavirus 2 (SARS-CoV-2) infection remains unequivocally unclear, it is possible that exposure to the virus may influence PC evolution by altering TMPRSS2 expression. This study aims to evaluate the long-term oncological outcomes of patients with metastatic PC who were undergoing medical therapy at the time of contracting SARS-CoV-2 and who resumed/continued anticancer treatment after recovery. **Methods:** We retrospectively evaluated a consecutive series of 151 metastatic PC patients who developed SARS-CoV-2 infection while receiving one active systemic anticancer therapy (125 metastatic castration-resistant PC (mCRPC) patients and 26 metastatic hormone-sensitive PC (mHSPC) patients). We evaluated variables that influence the ability to maintain or resume the ongoing therapy. For the maintained/resumed therapies, we calculated the post-infection overall survival (piOS) and the overall survival (OS). **Results:** Of the patients, 12.6% died due to SARS-CoV-2 infection, 10.6% recovered from the infection but failed to maintain/resume the ongoing anticancer treatment, and the remaining 76.8% maintained/resumed the treatment after recovery. Hospitalization, duration of infection, and the type of ongoing anticancer agent influenced these treatment changes. In the cohort of mCRPC patients, the median piOS was 32 months, and the median OS was 67.8 months. The median piOS was not achieved in the cohort of mHSPC patients, while the median OS was 122 months. The outcomes of single anticancer agents were in line with those of pivotal trials. **Conclusions:** Although observed in a highly selected population of PC patients who survived SARS-CoV-2 infection and were able to resume/maintain anticancer therapy, the survival outcomes of this study appear to be in line with those reported in pivotal studies, and SARS-CoV-2 infection does not seem to have adversely affected long-term oncological outcomes.

## Simple Summary

Although the relationship between androgen deprivation therapy for prostate cancer (PC) and the biological mechanisms of severe acute respiratory syndrome coronavirus 2 (SARS-CoV-2) infection remains unequivocally unclear, exposure to the virus may influence PC evolution by altering the expression of a specific gene. This study aims to evaluate the long-term oncological outcomes of patients with metastatic PC who were undergoing medical therapy at the time of contracting SARS-CoV-2 and who resumed/continued medical treatment after recovery. Our study suggests that the post-infection evolution of anticancer treatments was influenced by hospitalization, infection duration, and the type of ongoing anticancer agent. Moreover, among patients who were able to resume treatment, the survival outcomes we observed were within the range reported in historical data from pivotal clinical trials, suggesting that SARS-CoV-2 infection may not have led to a significant deterioration in long-term oncology outcomes. Our results provide further data on the complex relationship between PC and SARS-CoV-2 infection.

## 1. Introduction

Severe acute respiratory syndrome coronavirus 2 (SARS-CoV-2) infection was first detected in late 2019 in Wuhan, China [1], and quickly spread into a global pandemic of the new coronavirus disease 2019 (COVID-19).

Cancer was considered a significant risk factor for the severity of illness and/or mortality following exposure to SARS-CoV-2 [2], with cancer patients being more likely to require admission to an intensive care unit, mechanical ventilation, or to die [3].

This is primarily because the disease itself and cancer treatments can cause immunosuppression, thereby increasing the severity of the infection. Compared to the immunocompetent population, cancer patients are at a higher risk of dying from SARS-CoV-2 infection [4].

Moreover, among cancer patients, additional factors can negatively influence the infection outcomes, such as having a recent anticancer treatment [5], being younger [6], and having a diagnosis of chronic lymphocytic leukemia or prostate cancer (PC) [7].

Management of PC patients was dramatically disrupted during the pandemic, mainly in the presence of active medical therapy.

Cancer patients who tested positive for SARS-CoV-2 and were in critical condition due to the infection clearly had to be hospitalized, with the priority being to treat the infection rather than the cancer. The issue concerned managing those patients who had no symptoms or only minimal symptoms related to the infection.

Beyond androgen deprivation therapy (ADT), which is still the mainstay of PC medical management, from 2004 several life-prolonging agents (LPA) have produced a significant survival improvement in metastatic castration resistant PC (mCRPC) patients as well as in patients with metastatic hormone-sensitive PC (mHSPC): chemotherapeutics (docetaxel [8] and cabazitaxel [9]), androgen-receptor pathway inhibitors (ARPI: abiraterone [10,11,12,13], apalutamide [14], darolutamide [15,16], enzalutamide [17,18,19,20]), radiopharmaceuticals (Radium 223 [21] and Lu-PSMA [22]), and PARP inhibitors (olaparib [23]).

Regarding ADT, it was hypothesized during the pandemic that it might protect against severe SARS-CoV-2 infection by reducing TMPRSS2, a cellular target of the virus [24]. Regardless of this hypothesis, which was never unequivocally confirmed [25], prolonged-release pharmaceutical formulations do not prevent exposure to the drug in the event of a positive viral test result between doses. Furthermore, the toxicity profile has not raised concerns about continuing treatment during infection.

Although ARPI administration was considered safe in the presence of infection—and abiraterone was postulated to be capable of interfering with viral replication [26]—they were usually suspended for the duration of the infection. This was because it was thought that a brief suspension of ARPI treatment would not negatively affect disease control.

The situation is different when it comes to chemotherapy. This treatment can cause immunosuppression, which may negatively affect SARS-CoV-2 infection. Although there was no definitive evidence of a harmful association between chemotherapy and SARS-CoV-2 infection, it was suggested that anticancer treatment be interrupted, regardless of the presence of COVID-19 symptoms, and resumed once molecular tests were negative [27,28].

Once the infection had resolved, patients could resume any therapy that had been suspended.

Currently, no study has evaluated the long-term oncological outcomes of patients with metastatic PC who were undergoing anticancer medical therapy at the time of contracting SARS-CoV-2 and who resumed/maintained it after recovery.

This study aims to evaluate the long-term oncological outcomes of this population.

## 2. Materials and Methods

We retrospectively evaluated a consecutive series of metastatic PC patients who were receiving anticancer medical therapy at the time of SARS-CoV-2 onset between January 2020 and December 2022 in 10 Italian Hospitals.

For each patient, we reviewed the clinical charts and collected data concerning:

• PC history before infection: baseline characteristics (date of diagnosis, metastatic status at the diagnosis, Gleason score), type of medical treatments previously administered before SARS-CoV-2 infection (type of treatments), metastatic involvement at the time of infection, ongoing medical treatments at the time of infection (drug and start date);
• SARS-CoV-2 infection history: date of start (date of positive test), reason for diagnosis (symptoms, contact with infected people, screening for hospital access), hospitalization, end date (date of negative test), outcome (healing vs. death);
• PC history after infection: medical treatment resumption/maintenance (yes vs. no), date of disease progression after treatment resumption/maintenance, further medical treatments after progression, last follow-up date (date of last visit or death date), vital status (alive vs. dead).

### Statistics

Descriptive statistics were used to report patients’ characteristics: the median with interquartile range for continuous variables, and frequency (percentage) for categorical variables.

We compared baseline characteristics of patients with mCRPC and those with mHSPC according to the following variables: age, Gleason score, presence/absence of metastases at diagnosis, interval between PC diagnosis and SARS-CoV-2 infection onset, interval between the start of ongoing anticancer therapy and SARS-CoV-2 infection, and SARS-CoV-2 infection duration. We also evaluated the influence of several variables (age, hospitalization, disease status, interval from treatment start and infection, duration of infection, hospitalization) on treatment evolution (definitive interruption due to infection-related death, definitive interruption not related to death, resumption/maintenance) and overall survival (OS). The comparisons were made using the χ2 test for categorical variables and ANOVA for continuous variables.

Post-infection progression-free survival (piPFS) was calculated from the date of negative viral test results to the date of disease progression, death from any cause, or the date of last follow-up for censored patients.

Post-infection overall survival (piOS) was calculated from the date of negative viral test results to death from any cause or to the date of last follow-up for censored patients.

OS was calculated from the start date of ongoing anticancer medical agent until death from any cause, or the date of the last follow-up for censored patients.

piPFS, piOS, and OS were calculated only for patients who resumed or continued their previous medical therapy after recovering from the infection.

The Kaplan–Meier method was used to estimate the survival outcomes. Differences between groups categorized by variables of interest (type of anticancer agent, treatment line) were tested using the log-rank test.

Statistical analyses were performed with SPSS statistics software (version 25, http://spss.it/downloadv25 (accessed on 6 January 2026).

## 3. Results

We evaluated a consecutive series of 151 metastatic PC patients who developed SARS-CoV-2 infection while receiving one active systemic anticancer therapy.

The baseline characteristics of the patients are detailed in Table 1. The median age was 75 years (48–92). Most of the patients had a Gleason score ≥ 8 (62.9%) and were metastasis-free (47%) at the time of cancer diagnosis. At the time of SARS-CoV-2 infection, bone and nodal metastases were more frequently represented (76.8% and 61.6%, respectively).

Overall, 82.8% (125) of patients had mCRPC, and 17.2% (26) had mHSPC.

Higher Gleason score and metastatic involvement at the diagnosis were observed less frequently in mCRPC patients than in mHSPC patients (*p* = 0.04 and *p* = 0.002, respectively).

The median interval between the primary diagnosis of PC and SARS-CoV-2 infection diagnosis was 73.6 months (range 0.8–322.8) and, as expected, was significantly longer in mCRPC patients (82.1 months) than in mHSPC patients (28.3 months; *p* = 0.001).

Viral tests were mainly performed at the onset of symptoms (56.3%). In the remaining cases, the diagnosis was made through screening for hospital admission and contact with infected persons (21.8% in both cases). No statistically significant differences were observed between mCRPC and mHSPC populations.

At the time of infection onset, 34 patients (22.5%) were receiving ADT, 86 patients (56.9%) were receiving one ARPI, 26 patients (17.2%) were receiving one chemotherapeutic, and five patients (3.3%) were receiving another LPA. ADT was continued during the infection, while the other anticancer agents were stopped upon diagnosis of the infection.

The median duration of infection was 12 days (range 1–128) in the overall population and did not significantly differ between mCRPC patients (12 days) and mHSPC patients (8 days).

Hospitalization was necessary to manage infection symptoms in 47 cases; the hospitalization rate was similar in the mCRPC and mHSPC groups (31.2% and 30.7%, respectively).

Of the patients, 19 (12.6%) died due to SARS-CoV-2 infection. Sixteen (10.6%) recovered from the infection but failed to resume or maintain their ongoing anticancer treatment. The remaining 116 patients (76.8%) maintained or resumed treatment after recovery.

Hospitalization, infection duration, and type of ongoing anticancer agent predicted treatment evolution after recovery (see Table 2). Among the hospitalized group, only 51.1% of patients maintained or resumed the previous treatment; 34% died, and 14.9% recovered without resuming treatment; the respective figures for non-hospitalized patients were 88.5%, 2.9% and 8.7%, respectively (*p* < 0.0001). Patients who resumed or maintained their anticancer treatment had a mean infection duration of 16 days, compared with 23 and 43 days for those who were unable to resume or died (*p* = 0.009). A higher rate of treatment maintenance or resumption was observed among patients receiving ADT, one ARPI, or another LPA (79.4%, 81.4%, and 80%, respectively), compared with 57.7% among patients treated with chemotherapy (*p* = 0.001). Interestingly, we also observed a higher rate of treatment discontinuation after healing (34.6%) in the latter group, despite there being no excess mortality (7.7%).

### 3.1. mCRPC Population

The anticancer agents administered at the time of the SARS-CoV-2 infection development in the mCRPC population are detailed in Table 3. The most frequent treatment was one ARPI (63.2%), followed by ADT alone (16.8%) and chemotherapy (15.2%).

Most patients with mCRPC received first-line treatment (53.6%), consisting mainly of ARPI (92.5%). Twenty per cent received second-line treatment, most of which (40%) was also based on ARPI. The remaining patients were receiving more advanced treatment lines: in this setting, ADT, chemotherapeutic, ARPI, and other LPA were ongoing in 35.2%, 32.4%, 23.5%, and 8.8% of the patients, respectively. mCRPC patients who were receiving ADT alone in first-line have never been exposed to LPA. In contrast, those receiving ADT alone in subsequent lines were intended to receive it as a maintenance agent after completing one LPA and before starting a subsequent LPA line.

In Table 4, the evolution of anticancer treatments administered at the time of SARS-CoV-2 onset is detailed. Eighteen patients (14.4%) died due to SARS-CoV-2 infection, while 13 patients (10.4%) definitively discontinued the pre-infection ongoing anticancer treatment. Among these patients, 10 did not receive further anticancer treatments and died within five months from the time of infection recovery, two started a new anticancer therapy at the time of cancer progression, while the remaining patient, who had been receiving docetaxel before SARS-CoV-2 onset, switched to enzalutamide after infection recovery.

Of the 94 patients who maintained or resumed anticancer treatment active before SARS-CoV-2 infection, 53 experienced cancer progression and received additional LPA. Sixteen patients progressed but did not receive further anticancer treatment. Of the remaining 25 patients who did not experience disease progression, 20 were alive and receiving resumed therapy at the last study follow-up.

At a median follow-up of 24.9 months, in the overall cohort of mCRPC patients who maintained/resumed the pre-infection ongoing anticancer treatment, the median piPFS was 9.7 months (95% CI 5.4–13.9), the median piOS was 32 months (95% CI 20.4–43.6), and the median OS was 67.8 months (95% CI 54.7–80.8).

Clearly, there was a significant difference in outcomes by treatment line before SARS-CoV-2: patients on first-line treatment had longer median piPFS, median piOS, and median OS than patients on subsequent treatment lines (see Table 5).

In addition, patients treated with chemotherapy had a significantly shorter median OS (20.1 months) than those treated with either ADT (68.8 months) or one ARPI (median not reached) (*p* < 0.0001). At the multivariate analysis, the treatment type was the only variable that retained statistical significance. In particular, treatment with ADT and with ARPI increased the survival probability of 85% (HR 0.15; 95% CI 0.05–0.41) and 79% (HR 0.21 95% CI 0.06–0.67) compared to chemotherapy-based therapy.

At the time of infection, ARPI were mainly administered in first-line settings, where patients treated with abiraterone or enzalutamide showed median piPFS of 12.5 months (95% CI 4.3–20.8) and 23.5 months (95% CI 5.4–41.6), respectively, with no statistically significant difference. No statistically significant differences were found between the two drugs, also in terms of piOS and OS; median values were not reached in either case. The clinical history of all mCRPC patients who were receiving abiraterone or enzalutamide is graphically summarized in Figures S1 and S2.

At the onset of infection, patients were primarily receiving docetaxel as second-line therapy.

In this group of patients, we observed median piPFS, piOS, and OS of 1.8 months (95% CI 0.1–4.3), 18.1 months (95% CI 14.1–21.4), and 22.0 months (95% CI 17.1–27.8), respectively.

Most of the patients treated with cabazitaxel were receiving it in third- or later-line settings, with median piPFS, piOS, and OS of 3.0 months (95% CI 0.1–6.8), 16.4 months (95% CI 0.1–41.1), and 17.3 months (95% CI 0.1–35.1), respectively. The clinical history of all mCRPC patients who were receiving docetaxel or cabazitaxel is graphically summarized in Figures S3 and S4.

### 3.2. mHSPC Population

Table 6 details the anticancer agents administered to the mHSPC population at the time of SARS-CoV-2 infection development. By definition, all mHSPC patients were receiving a first-line anticancer treatment (otherwise they would have been considered to have mCRPC). The anticancer agents were represented by ADT alone in 13 patients (50%; 12 patients as exclusive treatment and one patient as maintenance after docetaxel + ADT), docetaxel + ADT in seven patients (26.9%), and ARPI + ADT in six patients (23.1%; five patients as exclusive treatment and one patient with darolutamide as maintenance after concurrent administration with docetaxel and ADT).

One patient (3.8%) died due to SARS-CoV-2 infection, and three patients (11.5%) definitively discontinued docetaxel treatment, which had been ongoing before SARS-CoV-2 onset, while maintaining ADT. The post-infection evolution of the active treatment at the time of SARS-CoV-2 infection onset is summarized in Table 7.

Of these patients, two did not receive further LPAs and are still alive in the absence of progressive cancer. One patient switched to abiraterone two months after recovering from SARS-CoV-2 infection and died 11.3 months later. Among the remaining 22 patients, four resumed the ongoing docetaxel + ADT and maintained ADT alone after the completion of the planned six chemotherapy courses. Three experienced a disease progression during the follow-up and received an ARPI, which was ongoing at the time of the present study. One is alive without disease progression and is still ongoing. One patient, who was receiving ADT after docetaxel completion, progressed during the follow-up, received an ARPI, and then died. Of the remaining 11 patients on ADT alone, four died (two after receiving an ARPI due to progression and two without experiencing progression), and seven were alive (three after receiving an LPA due to progressive disease and four still on ADT at the time of the study). Of the six patients receiving ARPI and ADT, one died without receiving another LPA after experiencing progression, and five were alive (four were still receiving ARPI treatment at the last follow-up, and one received another LPA after experiencing progression). The clinical history of all mHSPC patients is graphically summarized in Figure S5.

At a median follow-up of 24.8 months, the median piPFS was 17.4 months (95% CI: 9.8–25.0) in the overall cohort of mHSPC patients who maintained or resumed the pre-infection ongoing anticancer treatment. There were no statistically significant differences in piPFS between patients treated with ADT, ARPI + ADT, or docetaxel + ADT.

The median piOS was not achieved in mCSPC patients who resumed the pre-infection ongoing anticancer treatment: the projected 4-year survival rate was 59.8%. In this group of patients, median OS was 122 months (95% CI 47–197 months).

No statistically significant differences were observed in either piOS or OS according to treatment agents.

## 4. Discussion

To the best of our knowledge, this is the first paper to evaluate long-term oncologic outcomes in a population of metastatic PC patients who maintained/resumed the anticancer treatment that was active at the time of SARS-CoV-2 infection after its resolution. Our results suggested that the viral infection did not affect the long-term outcomes of these patients.

Overall, the management of patients with active cancer was dramatically impacted by the COVID pandemic.

Firstly, cancer patients were considered to be at high risk of developing more severe infections with a higher mortality rate than the non-cancer population. Several reports written during the pandemic described higher levels of severity and mortality rates among patients with active disease undergoing cancer treatment [5,6].

Moreover, among cancer patients, additional factors can negatively influence the infection outcomes, such as having a recent anticancer treatment [5], being younger [6], and having a diagnosis of chronic lymphocytic leukemia or PC [7].

During the pandemic, considerable attention has been paid to the outcomes of infection in PC patients treated with ADT, the cornerstone of medical management for PC.

In fact, it has been hypothesized that ADT may protect against severe infection by inhibiting TMPRSS2 expression, the SARS-CoV-2 target in host epithelial cells [24].

Nevertheless, due to heterogeneity and the lack of control for potentially critical factors influencing the relationship between infection severity and ADT, the various retrospective studies that have sought to clarify the issue have failed to confirm this hypothesis [25] unequivocally.

Although the protective role of ADT against infection severity remains unclear, exposure to the virus may influence PC evolution by altering TMPRSS2 expression.

Based on recently published data, TMPRSS2 gene expression is reduced acutely during infection but returns to normal levels after recovery [26].

Our data were obtained from a consecutive series of patients with metastatic PC, all of whom were treated with ADT alone or with an additional anticancer agent (mainly an ARPI), and who resumed/maintained this treatment after recovering from infection. Baseline characteristics were well balanced between the mCRPC and mHSPC populations, except for the rate of M1 cases at diagnosis, which was higher in the mHSPC group, and the interval between PC diagnosis and SARS-CoV-2 infection, which was longer in the mCRPC group. These findings were predictable given the well-known specific differences in disease history and clinical characteristics between these two clinical conditions. By definition, patients with mCRPC had a more extended clinical history and greater exposure to anticancer treatments than patients with mHSPC, and often had a higher disease burden.

In the mCRPC group, we observed a lower mortality rate (14.5%) than that reported in similar populations described at the beginning of the pandemic [27,28], reflecting the pandemic’s evolving landscape in terms of infection treatment and vaccine protection.

Compared with the results of pivotal trials, our data suggested that, in metastatic PC patients who were able to maintain or resume treatment ongoing before SARS-CoV-2 infection, this did not negatively affect their long-term oncological outcomes.

The addition of one ARPI, abiraterone or enzalutamide, to ADT in the chemo-naïve mCRPC setting significantly improved OS compared to ADT alone, with reductions in the risk of death of 19% and 23%, respectively [29,30]. In the COU-AA-302 trial, at a median follow-up of 49.2 months, patients who received abiraterone showed a median OS of 34.7 months [29], while in the PREVAIL trial, at a median follow-up of 31 months, the median OS was 35.3 months [30]. Our results, obtained in a highly selected population of PC patients surviving SARS-CoV-2 infection and able to resume/maintain anticancer therapy, seem to be in line with these data. With a median follow-up of 49.6 months for the abiraterone-treated group and 57.1 months for the enzalutamide-treated group, the median OS remained unreached in both groups.

Although docetaxel was the first approved LPA in the mCRPC first-line setting [8], after the introduction of ARPI in the same disease setting [29,30], it became the standard second-line treatment for patients who had developed resistance to abiraterone or enzalutamide, according to real-world practice.

According to a recently published network analysis of 22 studies evaluating the efficacy of second-line agents following first-line ARPI therapy, docetaxel is expected to achieve a median survival of 16.9 months in this setting [31] Similar results were observed in our study, in which the median OS from the start of docetaxel treatment was 22 months in patients who received docetaxel after ARPI.

The TROPIC study enrolled patients who had progressed on docetaxel and were treated with second-line cabazitaxel [9]. These patients showed a median OS of 15.1 months. In a more modern landscape, third-line cabazitaxel demonstrated a median survival of 13.6 months [32]. In our study, of the six patients who received cabazitaxel at the time of SARS-CoV-2 infection and resumed treatment, only one was on second-line therapy and had previously received only docetaxel; the others were on more advanced lines of treatment. Nevertheless, their survival time ranged from 7.3 to 29.9 months, with at least 17.4 months for half of them.

In pivotal clinical trials in mHSPC, adding an ARPI to ADT has typically resulted in a median OS of more than five years [12,13,14,16,19,20]. In our study, five patients were receiving ARPI therapy at the time of SARS-CoV-2 infection, and all of them resumed treatment after recovering. Of those patients, three were still receiving ARPI therapy at the time of the present study, after 42, 58, and 88 months, respectively. One patient experienced disease progression and was alive with a follow-up of 20 months. The remaining patient died 23 months after starting ARPI therapy.

The CHAARTED study demonstrated a significant improvement in OS in patients with high-volume mHSPC when docetaxel was added to ADT, compared with ADT alone, with a median OS of 51.2 months [33]. More recently, the ARASENS and PEACE1 studies demonstrated that adding an ARPI (abiraterone or darolutamide) to docetaxel and ADT was superior to docetaxel and ADT alone [15,34]. In our experience, four patients were receiving docetaxel and ADT at the time of viral infection and resumed treatment after recovery: they were all alive at the time of the present study, with follow-ups of 24, 26, 27, and 49 months, respectively. Another patient received darolutamide after docetaxel, as per the ARASENS study, and resumed treatment after infection: he was alive with a follow-up of 77 months.

Clearly, the present study shows several limitations. First of all, the lack of a comparison with a control group of metastatic PC patients who did not develop SARS-CoV-2 infection do not allow us to draw conclusive considerations concerning its impact on oncological outcomes in metastatic PC.

Moreover, because this study included only patients who survived infection and were able to recover/maintain therapy, its results can be applied only to a mPC patients infected with SARS-CoV-2 with a relatively better prognosis, who were sufficiently stable after infection and who are able to continue anti-cancer treatment.

We are then unable to provide detailed data on the infection history. We presented information concerning the infection duration as well as the need for hospitalization due to infection, while no data concerning its severity or treatment were available.

Given the limited number of patients available in our study for each group (except mCRPC patients treated with ARPI), it is challenging to compare the long-term oncologic outcomes observed in our study with those reported in the pivotal studies.

Finally, the retrospective nature of this study did not allow a case–control comparison between patients able to resume/maintain the anticancer treatments and those, which would reinforce the study findings.

In conclusion, in the selected patients who were able to resume treatment, the survival outcomes we observed were similar to the range of historical data reported in pivotal clinical trials, suggesting that SARS-CoV-2 infection may not have led to a significant deterioration in long-term oncology outcomes in these patients.

## 5. Conclusions

This study aimed to improve our understanding of the possible intricate and fascinating interplay between ADT and SARS-CoV-2 biology. Most of the available evidence was based on retrospective analyses of heterogeneous PC patient populations, exposed to ADT at different stages of disease and for varying durations. This evidence was unable to draw a definitive conclusion regarding the protective role of ADT in controlling the severity of infection [25]. Our findings suggest that SARS-CoV-2 infection did not negatively impact long-term oncological outcomes among patients who were able to maintain/resume their anticancer therapy after recovering from the illness.

## Figures and Tables

**Table 1:** Baseline characteristics of the patients in the overall population and in comparing mHSPC and mCRPC populations.

	All Patients	mHSPC	mCRPC	*p* Value
Median age (interquartile range)	75 (69–81)	75 (69–80)	75 (66–83)	NS
Gleason score				
<8	49 (32.5%)	4 (15.4%)	45 (36.0%)	0.04
≥8	94 (62.3%)	20 (76.9%)	74 (59.2%)	
Unknown	8 (5.3%)	2 (7.7%)	6 (4.8%)	
Stage at the diagnosis				
M0	71 (47.0%)	6 (23.1%)	65 (52.0%)	0.002
M1	63 (41.7%)	18 (69.2)	45 (36.0%)	
Unknown	17 (11.3%)	2 (7.7%)	15 (12.0%)	
Disease status at the time of COVID infection				
mCSPC	26 (17.2%)	26 (100.0%)	0 (0.0%)	NA
mCRPC	125 (82.8%)	0 (0.0%)	120 (100%)	
Site of metastases at the time of COVID infection				
Bones	116 (76.8%)	20 (76.9%)	96 (76.8%)	NS
Nodes	93 (61.6%)	18 (69.2%)	75 (60.0%)	NS
Liver	9 (6.0%)	2 (7.7%)	7 (5.6%)	NS
Lung	15 (9.9%)	5 (19.2%)	10 (8.0%)	NS

Legend: mCRPC = metastatic castration-resistant prostate cancer; mHSPC = metastatic hormone-sensitive prostate cancer; NA = not applicable; NS = not significant.

**Table 2:** Impact of hospitalization status and treatment type on treatment evolution.

	Permanent Discontinuation of Treatment Due to Death	Permanent Discontinuation of Treatment Not Due to Death	Treatment Maintenance/Resumption	Comparison Significance
	# of Patients (%)	# of Patients (%)	# of Patients (%)	
No hospitalization	3 (2.9%)	9 (8.7%)	92 (88.5%)	<0.0001
Hospitalization	16 (34%)	7 (14.9%)	24 (51.1%)	
ADT	7 (20.6%)	0	27 (79.4%)	0.001
ARPI	10 (11.6%)	6 (7.0%)	70 (81.4%)	
Chemo	2 (7.7%)	9 (34.6%)	15 (57.7%)	
Other LPA	0	1 (20%)	4 (80%)	

Legend: ADT = androgen deprivation therapy; ARPI = androgen receptor pathway inhibitor; Chemo = chemotherapeutic; LPA = life-prolonging agent.

**Table 3:** Ongoing anticancer treatment at the time of SARS-CoV-2 infection in the mCRPC population and their distribution by treatment line.

		Treatment Line	
Agent	First # (%)	Second # (%)	Third or More # (%)
ADT alone	3 (4.5) *	6 (24.0) **	12 (35.3) ***
Abiraterone + ADT	30 (45.5)	6 (24.0)	2 (5.9)
Cabazitaxel + ADT	0 (0.0)	1 (4.0)	10 (29.49)
Docetaxel + ADT	1 (1.5)	6 (24.0)	1 (2.9)
Ra223 + ADT	0 (0.0)	2 (8.0)	0 (0.0)
Olaparib + ADT	0 (0.0)	0 (0.0)	2 (5.9)
Lu-PSMA + ADT	0 (0.0)	0 (0.0)	1 (2.9)
ARSI + PARPi + ADT ****	1 (1.5)	0 (0.0)	0 (0.0)
Enzalutamide + ADT	31 (47.0)	3 (12.0)	6 (17.6)
Darolutamide + ADT ****	0 (0.0)	1 (4.0)	0 (0.0)

* no previous LPA, ** maintenance after first-line LPA, *** maintenance after second-line or subsequent treatment lines with LPA, **** treatment administered during a clinical trial.

**Table 4:** Post-infection evolution of anticancer treatments ongoing before SARS-CoV-2 infection in the mCRPC population.

	Anticancer Treatment Evolution After COVID-19 Infection		
	Permanent Discontinuation of Treatment Due to Death	Permanent Discontinuation of Treatment Not Due to Death	Treatment Maintenance/Resumption
Ongoing Anticancer Treatment at the Time of COVID Infection	# of Patients (%)	# of Patients (%)	# of Patients (%)
ADT alone	6 (28.6%)		15 (71.4%)
Abiraterone + ADT	4 (10.5%)	1 (2.6%)	33 (86.8%)
Cabazitaxel + ADT	1 (9.1%)	4 (36.4%)	6 (54.5%)
Docetaxel + ADT	1 (12.5%)	2 (25.0%)	5 (62.5%)
Ra223 + ADT		1 (50%)	1 (50%)
Olaparib + ADT			2 (100%)
Lu-PSMA + ADT			1 (100%)
ARSI + PARPi + ADT			1 (100%)
Enzalutamide + ADT	6 (15%)	5 (12.5%)	29 (72.5%)
Darolutamide + ADT			1 (100%)

**Table 5:** Survival outcomes in specific subgroups of mCRPC patients by treatment line and type of anticancer agent.

Patients	Median piPFS	Comparison Significance	Median piOS	Comparison Significance	Median OS	Comparison Significance
first-line treatment	18.2 (95% CI 10.0–26.4)	<0.0001	NR	<0.0001	NR	0.001
≥first-line treatment	3.6 (95% CI 1.8–5.3)		16.4 (95% CI 8.1–24.7)		35.1 (95% CI 10.5–59.6)	
ADT	58.3 (95% CI 31–385.4)	<0.0001	14.9 (95% CI 6.7–23.0)	<0.0001	68.8 (95% CI 47.2–90.3)	<0.0001
ARPI	43.3 (95% CI 28.2–58.6)		NR		NR	
Chemo	6.3 (5.3–7.2)		16.4 (95% CI 11.1–21.7)		20.1 (95% CI 12.9–27.3)	
First-line abiraterone	12.5 (95% CI 4.3–20.8)	NS	NR	NS	NR	NS
First-line enzalutamide	23.5 (95% CI 5.4–41.6)		NR		NR	
Second-line Docetaxel	1.8 (95% CI 0.1–4.3)		18.1 (95% CI 14.1–21.4)		22.0 (95% CI 17.1–27.8)	
≥third-line Cabazitaxel	3.0 (95% CI 0.1–6.8)		16.4 (95% CI 0.1–41.1)		17.3 (95% CI 0.1–35.1)	

Legend: CI = confidence interval; NR = not reached; NS = not significant.

**Table 6:** Ongoing anticancer treatment at the time of SARS-CoV-2 infection in the mHSPC population.

Treatment	# of Patients (%)
ADT alone	13 * (50%)
Abiraterone + ADT	1 (3.8%)
Docetaxel + ADT	7 (26.9%)
Apalutamide + ADT	2 (7.7%)
Enzalutamide + ADT	2 (7.7%)
Darolutamide + ADT (2)	1 ** (3.8%)

* 1 patient: ADT after DOC conclusion, ** 1 patient: Darolutamide after DOC conclusion.

**Table 7:** Post-infection evolution of anticancer treatments ongoing before SARS-CoV-2 infection in mHSPC population.

	Anticancer Treatment Evolution After COVID-19 Infection		
Ongoing Anticancer Treatment at the Time of COVID Infection	Permanent Discontinuation of Treatment Due to Death	Permanent Discontinuation of Treatment Not Due to Death	Treatment Maintenance/Resumption
	# of patients (%)	# of Patients (%)	# of Patients (%)
ADT alone	1 (7.7%)		12 (92.3%)
Abiraterone + ADT			1 (100%)
Docetaxel + ADT		3 (42.9%)	4 (57.1%)
Apalutamide + ADT			2 (100%)
Enzalutamide + ADT			2 (100%)
Darolutamide + ADT (2)			1 (100%)

## Data Availability

The original contributions presented in this study are included in the article/Supplementary Material. Further inquiries can be directed to the corresponding author.

## Supplementary Materials

The following supporting information can be downloaded at https://www.mdpi.com/article/10.3390/cancers18020264/s1, Figure S1: Alluvial plot describing the clinical history of mCRPC patients treated with abiraterone at the time of infection time; Figure S2: Alluvial plot describing the clinical history of mCRPC patients treated with enzalutamide at the time of infection time; Figure S3: Alluvial plot describing the clinical history of mCRPC patients treated with docetaxel at the time of infection time; Figure S4: Alluvial plot describing the clinical history of mCRPC patients treated with cabazitaxel at the time of infection time; Figure S5: Alluvial plot describing the clinical history of mHSPC patients.

## Abbreviations

The following abbreviations are used in this manuscript:

ADT	androgen deprivation therapy
LPA	life-prolonging agent
mCRPC	metastatic castration resistant prostate cancer
mHSPC	metastatic hormone-sensitive prostate cancer
PC	prostate cancer
piOS	post-infection overall survival
piPFS	post-infection progression-free survival
SARS-CoV-2	Severe acute respiratory syndrome coronavirus 2
