# Supplementary material for: Long-Term Oncological Outcomes in Metastatic Prostate Cancer Patients Who Are Able to Maintain/Recover Ongoing Anticancer Therapy After SARS-CoV-2 Infection—Results of the MEET-URO 22 Study
Source: Cancers (Basel). 2026 Jan 15;18(2):264. doi: 10.3390/cancers18020264 (PMC12838613; doi:10.3390/cancers18020264)

Figure S1: Alluvial plot describing the clinical history of mCRPC patients treated with abiraterone at the time of infection time

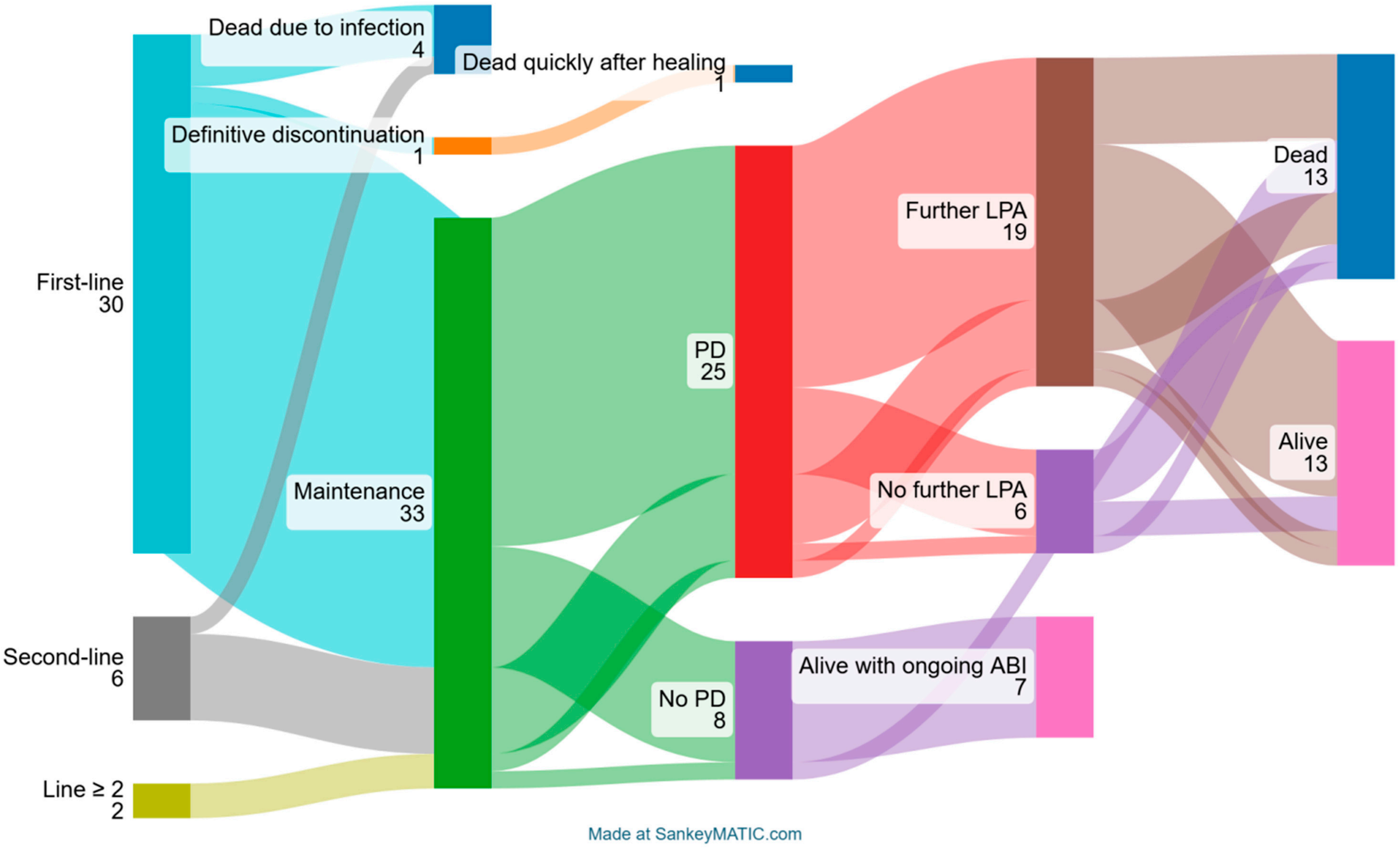

Figure S2: Alluvial plot describing the clinical history of mCRPC patients treated with enzalutamide at the time of infection time

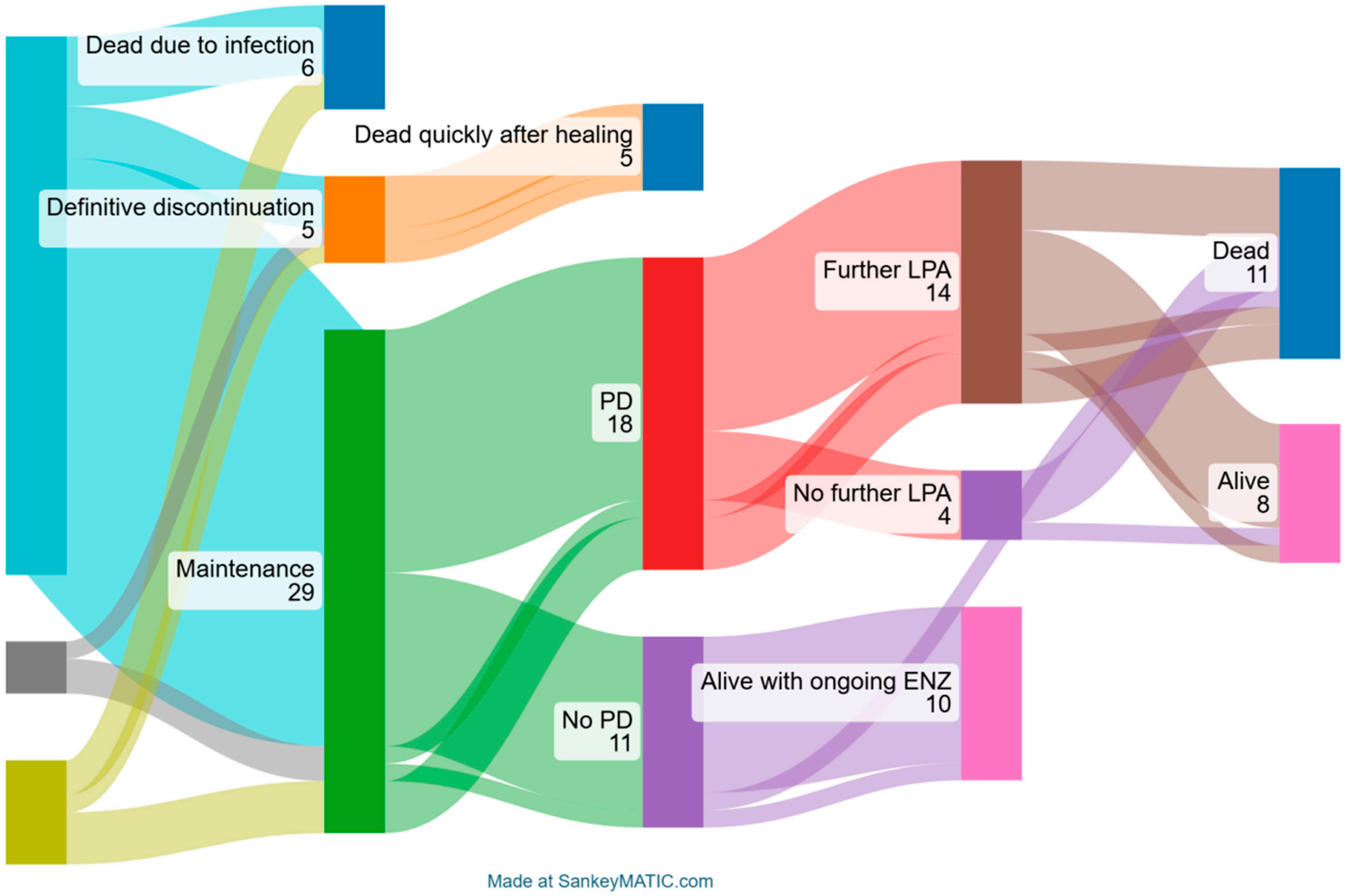

Figure S3: Alluvial plot describing the clinical history of mCRPC patients treated with docetaxel at the time of infection time

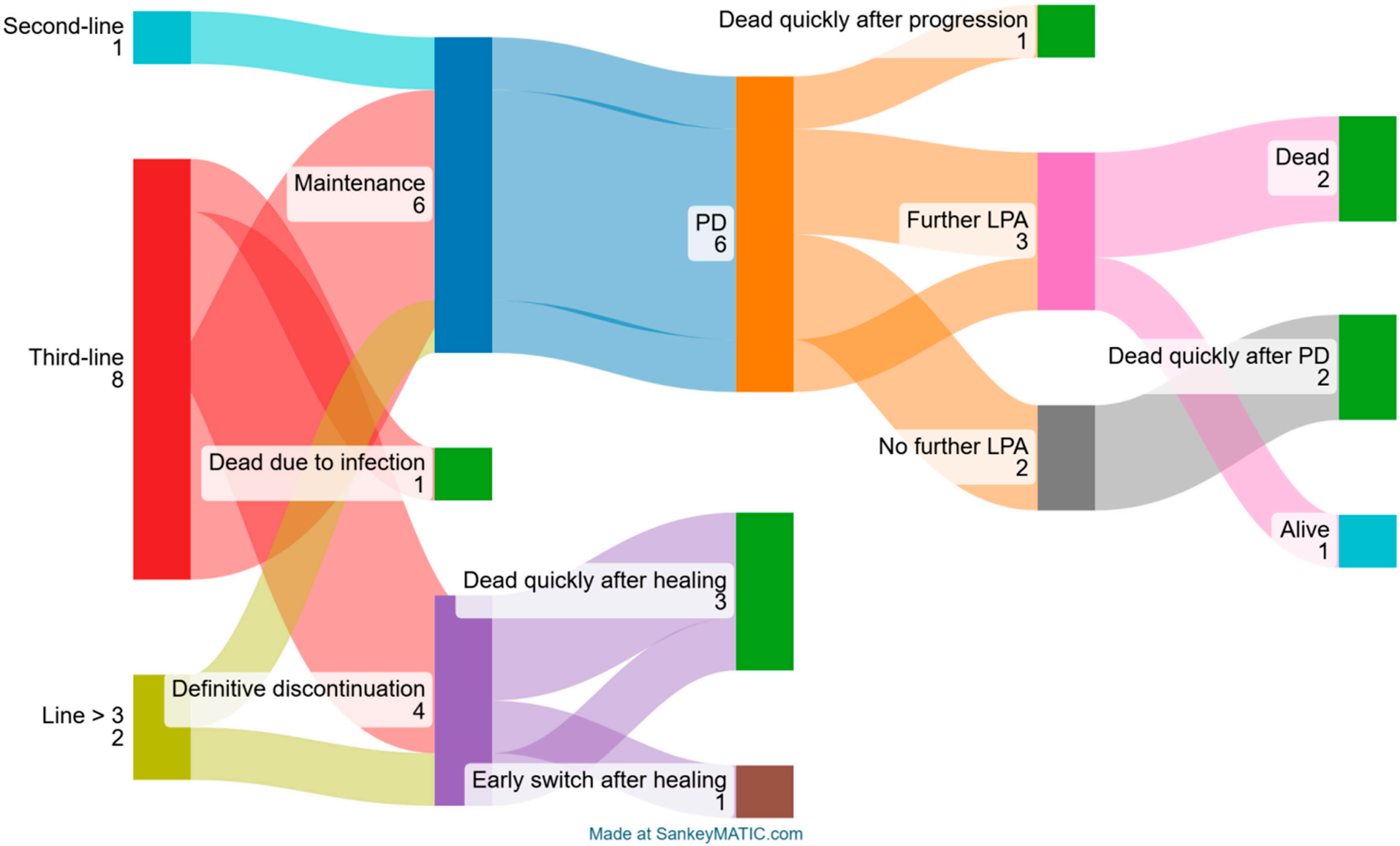

Figure S4: Alluvial plot describing the clinical history of mCRPC patients treated with cabazitaxel at the time of infection time

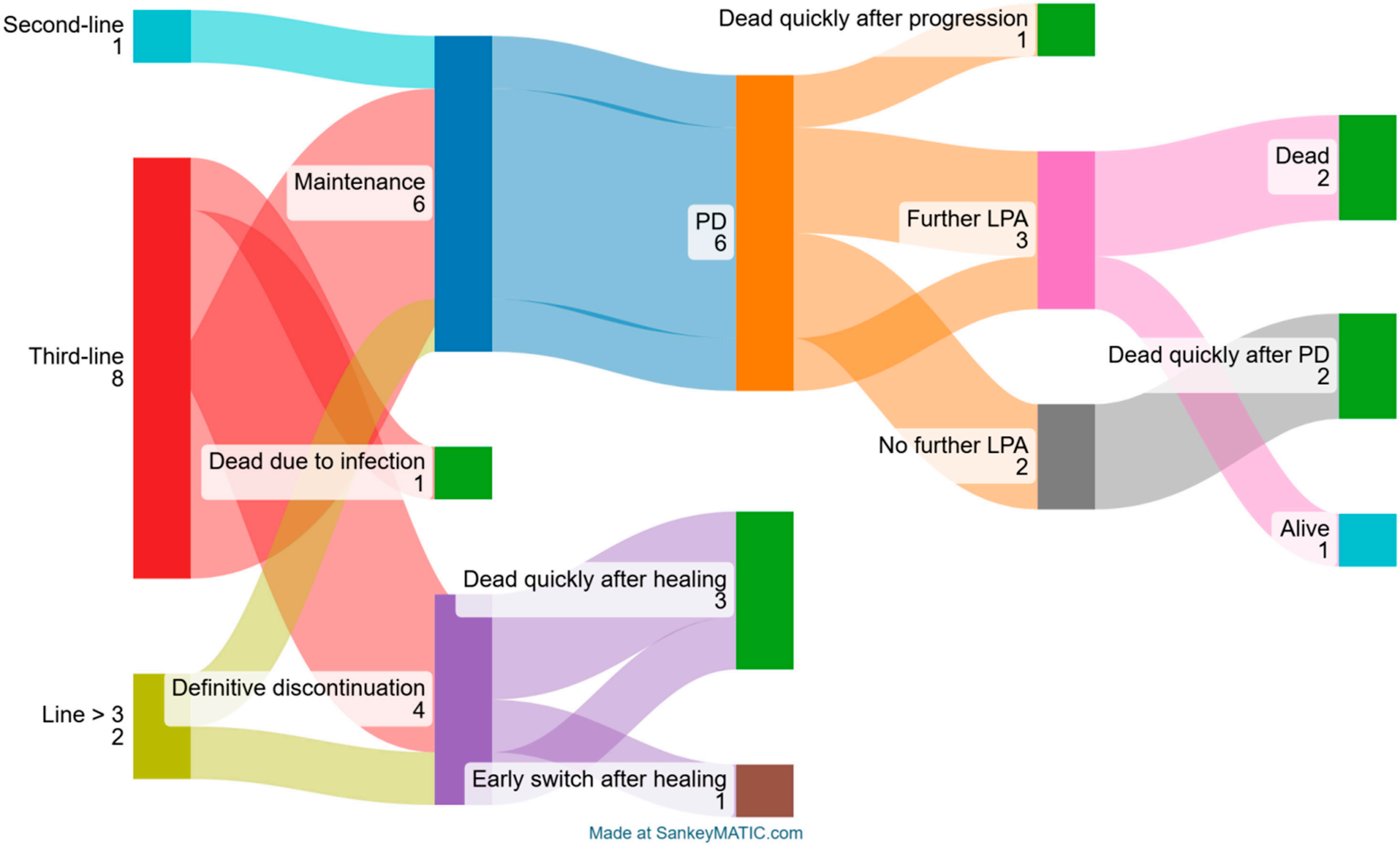

Figure S5: Alluvial plot describing the clinical history of mHSPC patients

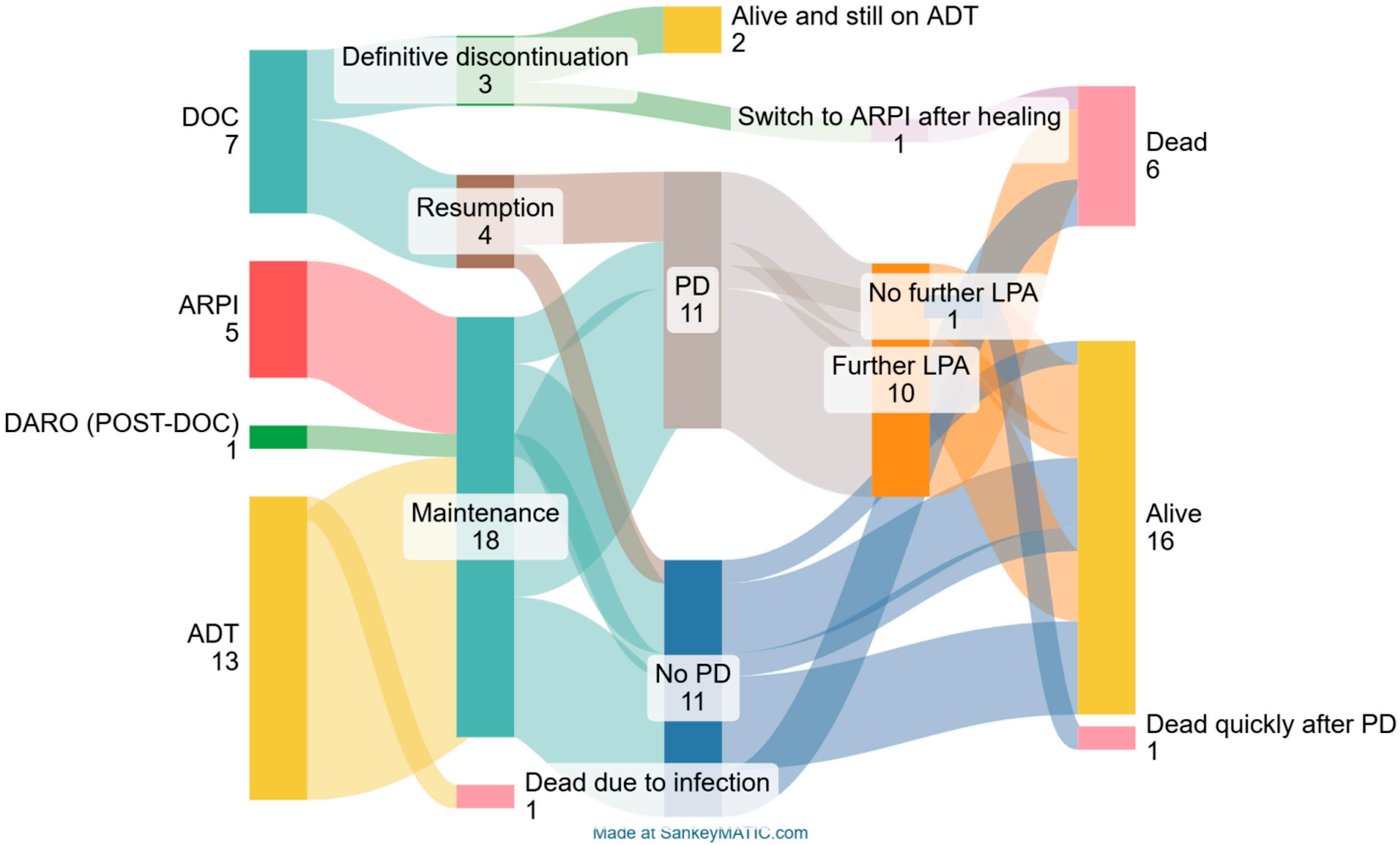

Supplement: Supplementary file 1 [file cancers-18-00264-s001.zip › cancers-3979415-supplementary.pdf]
